# Multimillijoule coherent terahertz bursts from picosecond laser-irradiated metal foils

**DOI:** 10.1073/pnas.1815256116

**Published:** 2019-02-13

**Authors:** Guoqian Liao, Yutong Li, Hao Liu, Graeme G. Scott, David Neely, Yihang Zhang, Baojun Zhu, Zhe Zhang, Chris Armstrong, Egle Zemaityte, Philip Bradford, Peter G. Huggard, Dean R. Rusby, Paul McKenna, Ceri M. Brenner, Nigel C. Woolsey, Weimin Wang, Zhengming Sheng, Jie Zhang

**Affiliations:** ^a^Key Laboratory for Laser Plasmas (Ministry of Education), School of Physics and Astronomy, Shanghai Jiao Tong University, Shanghai 200240, China;; ^b^Beijing National Laboratory for Condensed Matter Physics, Institute of Physics, Chinese Academy of Sciences, Beijing 100190, China;; ^c^Central Laser Facility, Science and Technology Facilities Council (STFC) Rutherford Appleton Laboratory (RAL), Didcot OX11 0QX, United Kingdom;; ^d^Collaborative Innovation Center of Inertial Fusion Sciences and Applications, Shanghai Jiao Tong University, Shanghai 200240, China;; ^e^School of Physical Sciences, University of Chinese Academy of Sciences, Beijing 100049, China;; ^f^Songshan Lake Materials Laboratory, Dongguan, Guangdong 523808, China;; ^g^Department of Physics Scottish Universities Physics Alliance (SUPA), University of Strathclyde, Glasgow G4 0NG, United Kingdom;; ^h^Department of Physics, York Plasma Institute, University of York, Heslington York YO10 5DD, United Kingdom;; ^i^RAL Space, STFC Rutherford Appleton Laboratory, Didcot OX11 0QX, United Kingdom;; ^j^Tsung-Dao Lee Institute, Shanghai Jiao Tong University, Shanghai 200240, China

**Keywords:** laser–plasma interaction, terahertz radiation, coherent transition radiation, extreme terahertz science

## Abstract

Ultrahigh-power terahertz (THz) radiation sources are essential for many applications, for example, THz-wave-based compact accelerators and THz control over matter. However, to date none of the THz sources reported, whether based upon large-scale accelerators or high-power lasers, have produced THz pulses with energies above the millijoule (mJ) level. Here, we report a substantial increase in THz pulse energy, as high as tens of mJ, generated by a high-intensity, picosecond laser pulse irradiating a metal foil. A further up-scaling of THz energy by a factor of ∼4 is observed when introducing preplasmas at the target-rear side. Experimental measurements and theoretical models identify the dominant THz generation mechanism to be coherent transition radiation, induced by the laser-accelerated energetic electron bunch escaping the target. Observation of THz-field-induced carrier multiplication in high-resistivity silicon is presented as a proof-of-concept application demonstration. Such an extremely high THz energy not only triggers various nonlinear dynamics in matter, but also opens up the research era of relativistic THz optics.

## Significance

Terahertz (THz) radiation, with frequencies spanning from 0.1 to 10 THz, has long been the most underdeveloped frequency band in electromagnetic waves, mainly due to the dearth of available high-power THz sources. Although the last decades have seen a surge of electronic and optical techniques for generating intense THz radiation, all THz sources reported until now have failed to produce above-millijoule (mJ) THz pulses. We present a THz source that enables a THz pulse energy up to tens of mJ, by using an intense laser pulse to irradiate a metal foil.

Terahertz (THz) radiation is usually utilized as a nonionizing probe in many science disciplines (1). With the recent advent of microjoule (μJ) THz pulses, THz radiation has started to serve as a mode-selective pumping driver for engineering particular transient states in materials (2–4). THz pulses, with energies over the millijoule (mJ) level and field strengths up to gigavolts per meter (GV/m), are expected to enable more intriguing applications such as, to name a few, ultrafast magnetic switch (5), compact THz electron accelerators and compressors (6, 7), THz-triggered chemistry (8), THz-assisted high-order harmonic generation (9), and multicolor single-shot THz bioimaging (10). Nevertheless, the generation of above-mJ THz pulses remains thus far a formidable challenge.

Currently, the most prominent approaches toward generating high-power THz pulses are based on conventional electron accelerators (11–13), and ultrafast laser systems (14). Although the former has output a THz pulse energy up to ∼600 μJ, the compromise between the electron bunch charge and bunch duration makes it difficult to enhance the THz energy. Via optical rectification, a maximum THz energy of 436 and 900 μJ has been generated from lithium niobate (LN) (15) and organic crystals (16, 17), respectively. Due to the inherent optical damage of crystals, one has to increase the size of both crystals and the pump spot for higher THz energy (18, 19). However, hurdles in the growth of large-size high-quality crystals and the inherent multiphoton absorption effect of crystals limit the potential of higher energy output in crystal-based THz sources.

Recently, laser-produced plasmas have attracted considerable interest as a damage-free medium for achieving compact intense THz sources (20, 21). Laser-driven gas-density plasmas usually deliver a few microjoules THz energy (22–25), which unfortunately saturates with increasing pump laser energy (26). Laser–solid interactions, by contrast, show advantages in the THz output energy, reaching hundreds of microjoules (27–32). The THz radiation generated usually has an ultrabroadband spectrum up to 30−100 THz, and the underlying THz generation mechanisms have not been fully understood. Gopal et al. (27) attribute the THz radiation produced at the rear side of thin solid targets to the target normal sheath acceleration of ions (33). On the other hand, transition radiation, induced by energetic electrons crossing the target–vacuum interface, also contributes (34), which has been proved indirectly by employing different target configurations (29).

In this report, utilizing an ultraintense, picosecond (ps) laser pulse to irradiate a metal foil, we demonstrate the efficient generation of low-frequency (<3 THz) coherent THz radiation with pulse energies exceeding multimillijoules, surpassing other THz sources (11–32). The direct manipulation of target-rear sheath fields with a controllable prepulse provides direct evidence that the THz radiation mainly originates from the coherent transition radiation induced by energetic electrons transiting the target surface, rather than the sheath acceleration, ions, or electrons confined in the sheath. A preliminary THz application experiment illustrates that even a small fraction of the generated THz radiation enables transient multiplication of carriers in semiconductors by several orders of magnitude on a picosecond time scale.

## Results

### THz Source.

**Fig. 1.**
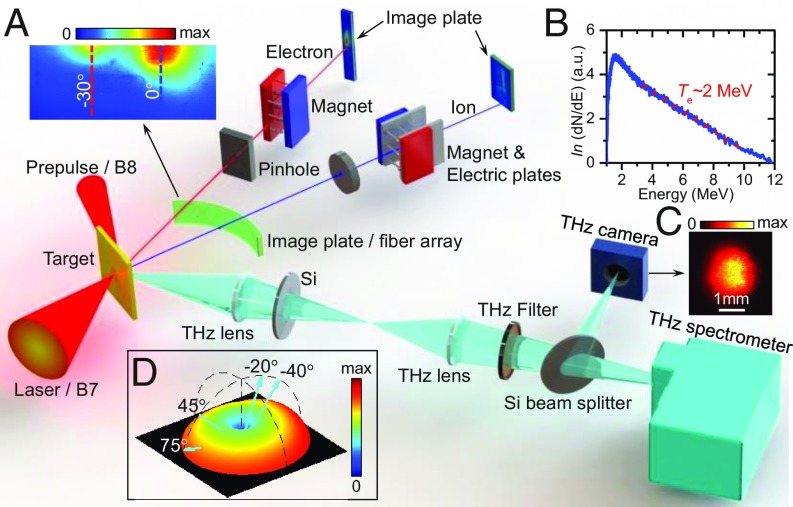
Schematic of the experimental setup. (*A*, *Insets*) Angular distribution of electrons measured with an image plate stack. (*B*) Measured electron energy spectrum and an exponential decay function curve fit. (*C*) Image of the THz spot measured with a CMOS-based THz camera. (*D*) Theoretically evaluated spatial distribution of THz radiation emitted from the target-rear surface, and cyan arrows indicating the THz detection directions. For clarity, only the THz detection at 75° with respect to the rear target normal is depicted, while similar configurations in other directions are not shown.

The experimental setup (see *Materials and Methods* for a detailed description) is shown in Fig. 1. A high-intensity picosecond laser pulse was focused onto a copper foil. Intense THz radiation, along with energetic ions and electrons, was emitted from the target-rear surface. To simultaneously characterize the accelerated ions (primarily protons) and escaping electrons in the same laser shots, THz lens systems with limited acceptance angles were used to collect the THz radiation in different directions.

**Fig. 2.**
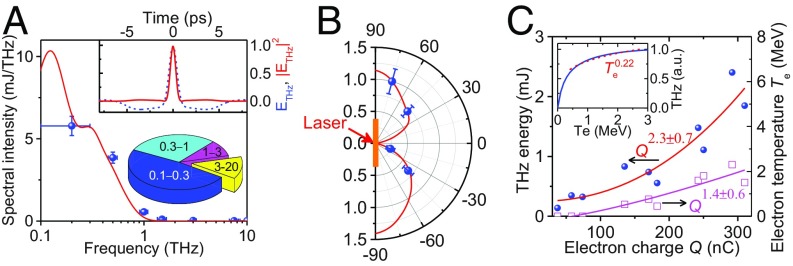
Characterization of THz radiation. (*A*) THz spectral distribution measured with band-pass filters (blue circles) and low-pass filters (*Inset*, *Lower Right*). Solid red curve shows the theoretically fitted spectrum with consideration of both the 1.5-ps divergent electron bunch and the 3-mm target size. (*Inset*, *Upper Right*) Normalized temporal profile of the THz field *E*_THz_ (blue dashed) and flux |*E*_THz_|^2^ (red solid), retrieved from the inverse Fourier transform of the theoretically fitted spectrum. (*B*) Measured (blue circles) and calculated (red curve) angular distributions of THz radiation in the detection plane. Data are normalized to unity at 75°. (*C*) Dependence of THz energy and electron temperature, *T*_e_, on the escaping electron charge, *Q*, measured when varying the pump laser energy. The data points of *T*_e_ = 0 correspond to the case in which the electron energy is lower than the detection limit (∼1 MeV) of the electron spectrometer. Curves are the power-law function fit. (*Inset*) Theoretically calculated transition radiation energy as a function of *T*_e_, and the power-law fit over 0.3−3 MeV (red dashed curve).

Intense THz radiation was observed. At a laser energy of ∼60 J, the THz energy in 0.12 steradian (sr) at 75° was measured to be ∼2.3 mJ at frequencies below 20 THz. Spectral measurements, either with a set of low-pass filters or narrowband band-pass filters, showed that the THz radiation was low-frequency (<3 THz) dominated (Fig. 2*A*). THz energy measurements at different directions showed that the THz radiation became weak near the rear target normal direction (Fig. 2*B*). By varying the laser energy, a power-law correlation of the THz energy to the electron charge was observed (Fig. 2*C*).

### THz Generation Mechanism.

**Fig. 3.**
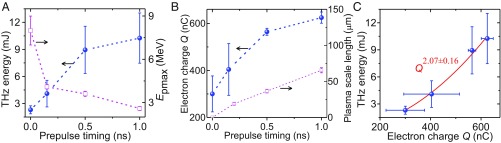
Identification of THz generation mechanism. (*A*) Dependence of spectrally integrated THz energy (blue circles) and the maximum proton energy (magenta squares) on the timing of the prepulse at the target rear. (*B*) Measured electron charge (blue circles) and simulated density scale length at the plasma–vacuum interface (magenta squares) as a function of the timing of prepulse. The error bar of plasma scale length is caused by the different laser intensity used in simulations. (*C*) Dependence of THz energy on the electron charge and a power-law function fit (red curve).

To identify the roles of sheath fields, ions, and electrons in the THz generation, we modified the sheath fields directly by controlling the preplasma at the target rear. An additional laser beam was introduced and focused to the target-rear surface, to generate an expanding preplasma ahead of the main pulse irradiating the front surface. The preplasma scale length was adjusted by varying the relative timing between the prepulse and main pulse. With increasing preplasma scale length, the maximum proton energy decreases significantly (Fig. 3*A*), resulting from the decrease in the sheath-field strength (35). By contrast, both the THz radiation and the electron charge measured increase significantly (Fig. 3 *A* and *B*). The distinctly different dependence of the THz radiation and sheath fields on preplasma scale length excludes the possibility that the sheath fields and ions contribute significantly to the measured THz radiation.

The coherent transition radiation (CTR) model (36, 37) can readily explain the THz radiation. The CTR energy, *W*_CTR_, generated by an electron bunch with a Boltzmann energy distribution, scales with the bunch charge *Q* and the electron temperature *T*_e_ approximately as *W*_CTR_ ∝*Q*^2^⋅$T_{\text{e}}^{0.22}$ (Fig. 2*C*, *Inset*). With increasing laser energy, both *Q* and *T*_e_ increase. According to the experimentally measured *T*_e_ ∝*Q*^1.4^^±^^0.6^, it is anticipated that *W*_CTR_ ∝*Q*^2.3^^±^^0.1^, which explains well the observed power-law index of 2.3 ± 0.7 (Fig. 2*C*). When introducing the target-rear preplasma, *T*_e_ does not vary much because the target-front electron acceleration is not affected. Hydrodynamic simulations with the MULTI−fs code (38) show that, the preplasma density remains comparably high for the low-frequency THz radiation in the <1-ns evolution time scale (39), and the density scale length at the plasma–vacuum interface remains much less than the THz wavelength (Fig. 3*B*). In this case, modeling the target-rear surface as a sharp metallic boundary is a good approximation (22, 37, 40), and the conventional CTR scenario is still applicable. This is also suggested by the quadratic dependency shown in Fig. 3*C*. Based on the measured angular distribution and energy spectra of electrons (Fig. 1 *A* and *B*, *Insets*), one can calculate the radiation spectrum and angular distribution from the CTR theory (*Materials and Methods*). The calculated values agree well with the measurements (Fig. 2 *A* and *B*).

**Fig. 4.**
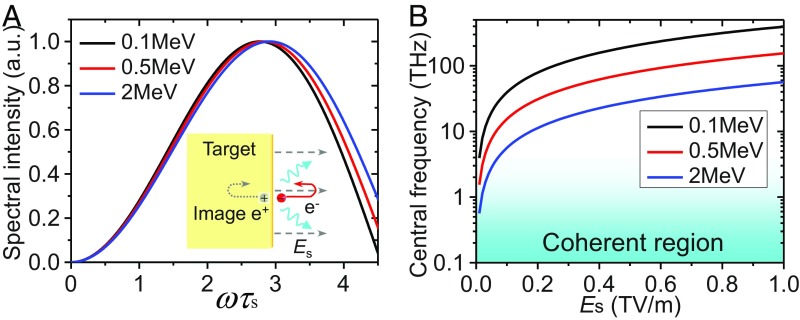
Radiation of electrons confined in the sheath. The electron kinetic energy is indicated. (*A*) Calculated normalized radiation spectra generated by a single electron. (*Inset*) Schematic illustrating the scenario where an electron crosses the target surface, and returns back under the action of the sheath field. The transient electric dipole consisting of the electron and its image charge emits electromagnetic radiation. (*B*) Central radiation frequency as a function of the sheath-field strength, *E*_s_. The cyan area sketches the coherent frequency region for a picosecond electron bunch.

One may wonder whether those low-energy electrons, which are dragged back to the target by the strong sheath field, are capable of forming secondary transition radiation and thus contributing to the total THz radiation. Our experimental results (Fig. 3) answer this question: the electrons confined at the vicinity of the target contribute little to the THz transition radiation. A simplified analytic model is proposed to understand this (*Materials and Methods*). As shown in Fig. 4*A*, the radiation spectrum, *I*_e_(ω), emitted by a single electron in the sheath, depends mainly on the lifetime of electrons in the sheath, τ_s_, which is determined by the electron kinetic energy and sheath-field strength. The coherent radiation intensity, *I*_b_(ω), generated by an electron bunch, is approximately given as *I*_b_(ω) ∝*F*^2^(ω)⋅*I*_e_(ω), where *F*(ω) is the electron bunch form factor. For the picosecond electron bunch in our picosecond laser experiment, *F*(ω) gets rather weak at the frequency region over 1 THz, overlapping little with *I*_e_(ω) especially at high sheath fields (Fig. 4*B*), and hence those electrons confined in the sheath cannot efficiently generate coherent radiation below 1 THz, but possibly contribute to the weak high-frequency radiation observed in the experiment.

Qualitatively, the adverse role of sheath fields in the CTR can be understood by analogy to the formation-zone effect (41), where the backward radiation generated by electrons reentering the target interferes destructively with the forward radiation generated by electrons exiting the target surface, thus suppressing the total forward THz radiation. When the target-rear prepulse is on, the preplasma reduces the sheath-field strength (35), fewer electrons are dragged back to the target, resulting in weaker destructive interference, and meanwhile more electrons escape from the target (39), which more efficiently produces stronger THz radiation (Fig. 3).

### High-Field THz Pump Experiment.

**Fig. 5.**
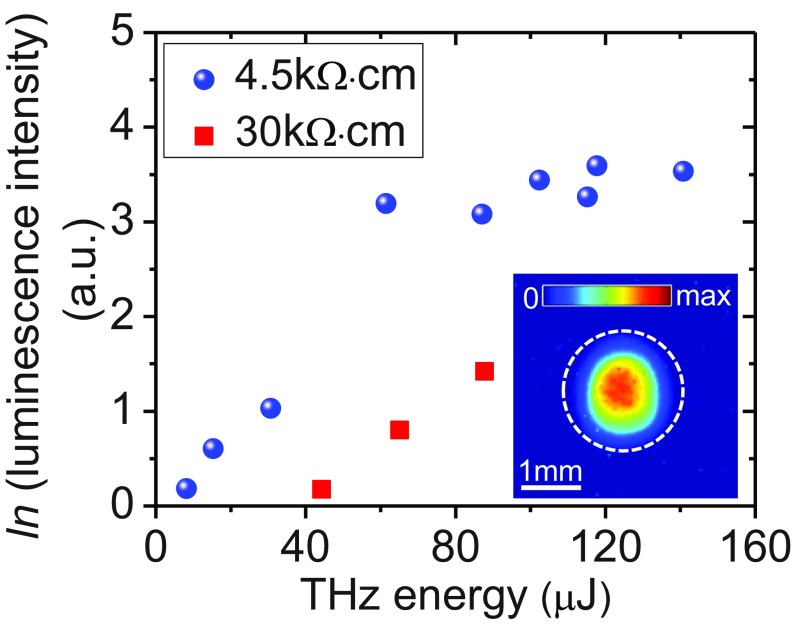
THz field-induced luminescence intensity, emitted from the Si samples with a resistivity of 4.5 kΩ⋅cm (blue circles) and 30 kΩ⋅cm (red squares), respectively, as a function of THz energy. Note that the luminescence intensity is given in natural logarithmic scale. (*Inset*) A typical luminescence image.

A fraction of the THz radiation was focused onto high-resistivity silicon (Si) wafers. If the THz field is adequately high, the interband luminescence will emerge (42) despite the band gap (1.12 eV) of Si exceeding the central THz photon energy by a factor of ∼1,000. A scientific-grade camera was used to observe the luminescence emitted from the Si samples. Bright near-infrared luminescence was observed within the THz focal spot. The Si wafer with a lower resistivity exhibits brighter luminescence. The luminescence intensity first increases exponentially with the THz energy, and then saturates when the THz energy on the Si sample is over ∼100 μJ (Fig. 5) with an estimated field strength of ∼0.08 GV/m at a spot size of ∼3 mm.

The presence of observable luminescence from high-purity Si implies a substantial multiplication in the number of interband electron–hole (*e*-*h*) pairs. We attribute the generation of massive *e*-*h* carriers mainly to the process of impact ionization (43), where an energetic conduction-band electron collides with a valence-band electron, creating two conduction electrons and a hole. Since the electric field required to ionize the phosphorus donor in Si is only ∼180 kV/cm (44), the impurity donors can be ionized fully on the rising edge of THz pulses, and subsequently are accelerated by the THz field. If an electron gains a kinetic energy exceeding the band gap, impact ionization will occur efficiently (45). After each impact ionization event, the carrier number is doubled, and the original electron and the newly born *e*-*h* pair can regain energy from the THz field in a cascade way. Such an avalanche-like ionization behavior will lead to the carrier density increasing exponentially over time and with THz pulse energy. This fully explains the experimentally observed nonlinear behavior of luminescence intensity with the THz energy. On the other hand, the generated high-density carriers enhance the carrier-phonon scattering and the Coulomb scattering among carriers, which will in turn reduce the energy of carriers and suppress impact ionization (43). This may account for the saturation of luminescence observed at high THz energies.

Another mechanism that possibly contributes to the carrier generation at high THz fields is Zener tunneling (42). According to the interband tunneling model developed by Kane (46), the density of carriers generated via field-induced tunneling is estimated to be only ∼8 × 10^5^ cm^−3^ under the bias of 0.08-GV/m THz field lasting 1.5 ps, much less than the impurity concentration in samples. Hence Zener tunneling contributes little to the carrier generation in our experiment.

## Discussion

**Fig. 6.**
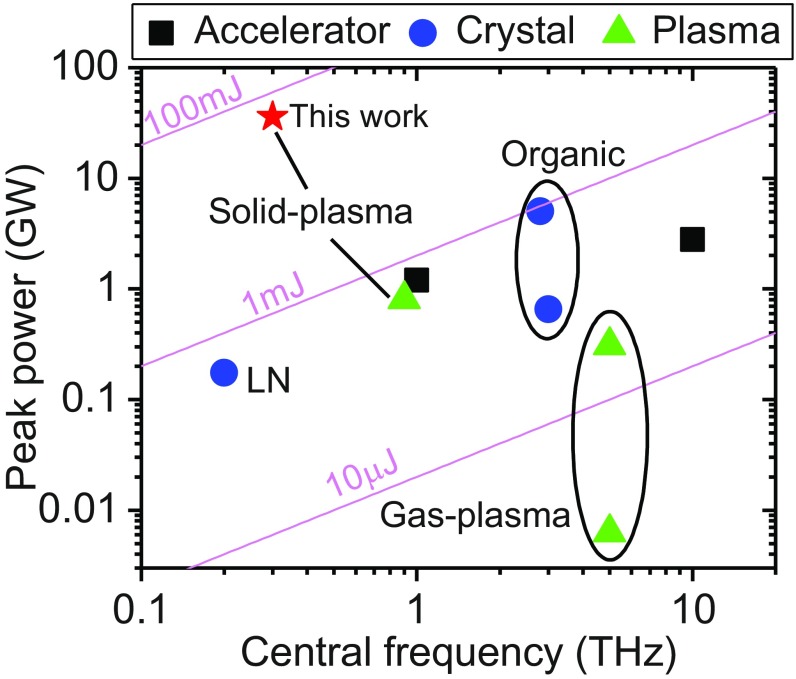
Comparison of currently available high-peak-power THz sources. The data are referenced from previously reported typical results of THz sources based on conventional accelerators (12, 13) (black squares), optical rectification from crystals (blue circles) like LN (15) and organic crystals (16, 17), and gas (24, 25)/solid–density plasmas (27) (green triangles). The red star represents the data presented in this paper. Magenta curves represent different energy ranges for half-cycle THz pulses.

Given the good agreement of THz measurements with the CTR model, one can evaluate the total THz pulse energy by extrapolating experimental measurements with model calculations (*Materials and Methods*). At a pump laser energy of ∼60 J in the case without target-rear prepulses, the total energy of THz pulses emitted from the target rear is determined to be ∼55 mJ (±20%) within the frequency range up to 3 THz. This corresponds to a laser-THz energy conversion efficiency of ∼0.1% and a peak power of ∼36 GW (Fig. 6) for the pulse duration of ∼1.5 ps (see Fig. 2*A* for the retrieved quasi–half-cycle THz waveform). The parameters evaluated above can be even higher since the THz energy can be further boosted by the target-rear preplasma. For the case in which the THz energy is increased by a factor of ∼4 (Fig. 3*A*), the THz electric field, *E*_THz_, at the target-rear surface is estimated to be ∼4 GV/m (*Materials and Methods*). Although a higher THz peak field of 8.3 GV/m has been reported at a multi-THz central frequency and thus in a much smaller focal spot (17), a comparably low central frequency of ∼0.3 THz here leads to a much higher ponderomotive potential *U*_p_ ∼200 keV. The normalized vector potential, as a critical parameter to characterize the electromagnetic field strength, is estimated as *a*_0_ = *eE*_THz_/*m*_e_*cω*_0_ ∼1.2, where ω_0_ is the central angular frequency, *e* and *m*_e_ are the charge and mass of the electron, respectively, and *c* is the speed of light. This already arrives at the realm of relativistic optics, which was not accessible on a half-cycle time scale previously.

Given that the THz radiation is emitted in a rather large divergence angle (Fig. 2*B*), one needs to use collection optics with large acceptance angles to deliver more available THz energy for practical applications. For example, if an ellipsoidal mirror with an acceptance solid angle of ∼4 sr was applied (27, 32), up to ∼70% of the total THz energy would be collected.

In addition to a high THz energy, our experimental results also present approaches to tuning laser-driven CTR-based THz sources. On the one hand, transferring the target-rear preplasma scheme to femtosecond laser systems, one may efficiently obtain millijoule-level THz pulses by adopting hundreds-of-millijoule tabletop femtosecond lasers, which can be operated in a high repetition rate (typically 10 Hz) and be available in university-scale laboratories. On the other hand, by comparing the broadband THz spectra (up to ∼30 THz) obtained previously in femtosecond laser-driven cases (29–32) with that in our current picosecond laser experiment, it is inferred that the THz spectra can be tunable by varying the pump laser pulse duration.

In conclusion, we have experimentally demonstrated the efficient generation of low-frequency THz pulses with an ultrahigh energy of ∼50 mJ, exceeding other state-of-the-art THz sources by nearly one order of magnitude. A further THz energy boost is obtained by introducing preplasmas to enhance the number of electrons escaping the sheath field generated at the target-rear surface. The application potential of the THz source is illustrated with a preliminary THz pump experiment, where a strong-field THz pulse induces the extraordinary multiplication of carriers in semiconductors. Multimillijoule THz sources reported here could enable the study of relativistic optics in the THz regime. Together with intrinsically synchronized energetic particles and photons generated concomitantly in laser–plasma interactions, more opportunities in the extreme THz science (47) will be opened up via multifunctional pump–probe experiments.

## Materials and Methods

### Laser System.

The experiment was carried out at the Rutherford Appleton Laboratory using the Vulcan laser (48) operating in a dual-laser beam (B7 and B8) configuration. The beam B7 (∼1.5-ps pulse duration, ∼1,053-nm central wavelength) as the main pump pulse, was focused by an f/3 off-axis parabolic (OAP) mirror onto the 100-μm-thick copper (Cu) foil target at an incidence angle of 30° and an ∼5-μm focal spot size (full width at half maximum, FWHM). For the maximum laser energy of ∼60 J on target, the peak laser intensity was ∼5 × 10^19^ W/cm^2^ (*a*_0_ ∼ 6). The beam B8 (5 ± 2 J, ∼10-ps pulse duration) as the prepulse, was focused by an f/15 OAP mirror onto the target-rear surface at an incidence angle of 75° and an enlarged focal spot size of ∼0.5 mm × 1.9 mm, corresponding to a laser intensity in the range of ∼(3−6) × 10^13^ W/cm^2^. The fairly large spot size of B8 produced a quasi–one-dimensional evolution of the preplasma, and ensured full overlap of the preplasmas with the B7-accelerated electron bunch. Neither THz radiation nor particle acceleration was detected when only B8 was applied on the target. Before the arrival of B7 at the target, the target-front surface was not perturbed by the target-rear prepulse. Evidences of this came from the fact that the target-front electron and optical diagnostic signals did not vary with the prepulse.

### THz Characterization.

The THz radiation at 75°, 45°, −20°, and −40° (Fig. 1*D*, *Inset*) with respect to the rear target normal was collected with similar lens configurations, and then relayed into THz energy and spectrum detection systems. To remove radiation at other wavelengths and avoid saturation of the THz detectors, THz filters and high-resistivity Si wafers were placed in the THz path. Owing to the high energies of the THz pulses produced in the experiment, a filter-based THz spectrometer was applied to measure the discretized THz spectrum in a single shot. In the THz spectrometer, the THz beam was splitted into eight beamlets. Low-pass or narrowband band-pass THz filters with varying cutoff or central frequencies were inserted in different beamlet paths, and the filtered THz radiation was measured by cross-calibrated pyroelectric detectors. The THz spectral intensity is retrieved as *I*_THz_ = *S*_det_/(*R*_det_⋅*T*_m_⋅Δω), where *S*_det_ is the detector signal, *R*_det_ is the detector responsivity averaged over the transmission bandwidth, Δω, of band-pass filters, and *T*_m_ is the overall transmittance of THz components in the path, respectively. The spectrally integrated THz energy is obtained as *W*_THz_ = *S*_det_/(*R*_ave_⋅*T*_m_), where *R*_ave_ is the average responsivity within 10 THz. The inverse Fourier transform of the fitted THz spectrum (Fig. 2*A*, *Inset*) shows ∼83% of the total energy is distributed in a pulse duration of τ_THz_ = 1.5 ps.

A complementary metal-oxide-semiconductor (CMOS)-based camera (DataRay Inc.) was used to image the THz source (Fig. 1*C*, *Inset*), but it only highlighted the high-frequency components because of its decreasing sensitivity with decreasing radiation frequencies. Since the transverse formation length (37) of sub-THz CTR induced by a megaelectron bunch is greater than the finite target size (∼3 mm) used in the experiment, the THz generation area, *A*_THz_, is limited to the target size. Eventually, the peak THz electric field at the target surface is estimated as $E_{\text{THz}}=\sqrt{2W_{\text{THz}}/c\varepsilon_{0}\tau_{\text{THz}}A_{\text{THz}}}$. Because a 1:1 lens-imaging system was adopted to collect and focus a small part of THz radiation onto the sample, the THz spot on the sample was assumed to be similar to the THz source at the target surface.

### Electron and Ion Characterization.

Three diagnostic methods were used to characterize the escaping electrons. An electron spectrometer, consisting of a permanent magnet with field strengths of 0.1 T and an image plate (IP) detector, was used to detect the energy spectrum of electrons. The electron temperature was measured to be ∼2 MeV (Fig. 1*B*, *Inset*) at the pump laser energy of ∼60 J. In some shots a four-layered IP stack (49), positioned 5 mm below the horizontal plane, was employed to record the spatial distribution of the electron bunch (Fig. 1*A*, *Inset*), showing a double-peak angular distribution with a divergence angle of ∼30°. A Cherenkov fiber array (50) was run every shot to monitor the change of the electron number in different directions. By calibration with IP, the total electron charge was obtained by integrating the signal from each fiber loop with consideration of the correction factor. The energy spectrum of ions was measured with a Thomson parabola spectrometer placed along the rear target normal.

### Modeling Radiation Induced by Electrons in the Sheath.

Given the fact that both the lifetime and traveling distance of low-energy electrons in the sheath are quite small compared with those of sheath evolution, the sheath field, *E*_s_, can be considered approximately to be uniform along the rear target normal for a specific electron. An electron leaves orthogonally the target surface with a momentum of *p* at *t* = 0, and returns back to the target at *t* = 2τ_s_, where τ_s_ = *p*/*eE*_s_. By adopting a model of transient electric dipole consisting of the electron and its image charge, the resulting radiation spectral intensity is deduced as

$$I_{e}=\frac{e^{2}}{4\pi^{3}\varepsilon_{0}c}\sin^{2}\theta {\left|G_{e}\left(\omega \tau_{s},p/m_{e}c,\theta \right)\right|}^{2},$$ [1]

where $G_{e}\left(\omega ,p,\theta \right)=\omega \int_{-1}^{1}d\zeta e^{i\omega \zeta }p\zeta /\sqrt{1+p^{2}\zeta^{2}}\cos\left\{\frac{\omega }{p}\left[\sqrt{1+p^{2}}-\sqrt{1+p^{2}\zeta^{2}}\right]\cos\theta \right\}$, *θ* is the angle between the electron direction and the radiation wave vector.

### Calculation of CTR.

For an electron bunch crossing an ideal conductor–vacuum interface, the CTR energy, *W*_CTR_, emitted per unit angular frequency *dω* and unit solid angle *d*Ω can be expressed as

$$W_{CTR}\propto Q^{2}{\left|\int d^{3}pg\left(p\right)\xi F\left(\omega \right)\cdot D\right|}^{2},$$ [2]

where *Q* is the electron bunch charge, *g*(***p***) is the momentum distribution function, which can be decomposed into two parts, the energy spectrum and the angular distribution. ξ is the normalized amplitude of radiation electric fields. *D* is the correction factor due to the finite target size. Expressions for ξ and *D* can be found in ref. 37. *F*(ω) is the bunch form factor, defined as the Fourier transform of the normalized electron bunch temporal profile. For a collimated electron bunch with a Gaussian temporal distribution, *F*(ω) = exp(−ω^2^ $\tau_{b}^{2}$/2), where τ_b_ is the root-mean-square bunch duration. For a divergent electron bunch, the radiation spectrum will be broadened (36, 37).

Typically the electron bunch duration is on the order of the laser pulse duration (FWHM∼1.5 ps in this experiment). By substituting the measured spatial and energy spectral distributions (Fig. 1 *A* and *B*) of the electron bunch into Eq. **2**, one can numerically calculate the 3D spatial distribution of CTR (Fig. 1*D*, *Inset*). Spatially integral of the calculated angular distribution indicates that the total energy is ∼28 times the energy in 0.12 sr at 75°, which is measured to be 1.96 mJ (±20%) within 3 THz in the experiment. Hence the total THz energy emitted from the target-rear surface is evaluated to be ∼55 mJ.

### Data Availability.

Data associated with research published in this paper can be accessed at https://edata.stfc.ac.uk/handle/edata/747.
